# The characteristics of the implicit body model of the trunk

**DOI:** 10.1177/03010066241248120

**Published:** 2024-05-05

**Authors:** Simon Pratt, Benedict M. Wand, Dana A. Hince, Mervyn J. Travers, Lee Schneider, Sara Kelly, William Gibson

**Affiliations:** 3431 The University of Notre Dame Australia, Australia

**Keywords:** body model, body representation, midline perception, width perception, height perception

## Abstract

Knowing where the body is in space requires reference to a stored model of the size and shape of body parts, termed the body model. This study sought to investigate the characteristics of the implicit body model of the trunk by assessing the position sense of midline and lateral body landmarks. Sixty-nine healthy participants localised midline and lateral body landmarks on their thorax, waist and hips, with perceived positions of these landmarks compared to actual positions. This study demonstrates evidence of a significant distortion of the implicit body model of the trunk, presenting as a squatter trunk, wider at the waist and hips. A significant difference was found between perceived and actual location in the horizontal (*x*) and vertical (*y*) directions for the majority of trunk landmarks. Evidence of a rightward bias was noted in the perception of six of the nine body landmarks in the horizontal (*x*) direction, including all midline levels. In the vertical (*y*) direction, a substantial inferior bias was evident at the thorax and waist. The implicit body model of the trunk is shown to be distorted, with the lumbar spine (waist-to-hip region) held to be shorter and wider than reality.

*Body representation* incorporates the prediction, construction and evaluation of one's body structure (Longo & Haggard, 2010; Pazzaglia & Zantedeschi, 2016). Our body representations are influenced by tactile information, proprioceptive senses providing knowledge of location and posture of our limbs, visual inputs regarding how we appear and where we are in space, auditory signals (Azañón et al., 2016; Longo et al., 2010), inputs related to cultural, conceptual and linguistic aspects (e.g., the functional purpose) of body parts (de Vignemont, 2010) and cognitive elements, incorporating experiences and formal knowledge, thoughts and emotions (de Vignemont, 2010; Levenig et al., 2016). Three broad types of body representation have been proposed: body model, body schema and body image (Longo, 2015a). There is debate regarding the exact boundary of each (Berlucchi & Aglioti, 2010; de Vignemont, 2010; Longo, 2016), with these representations interacting and influencing one another (Giummarra et al., 2008; Longo, 2015a).

Understanding the moment-by-moment position of the body in space is a good example of how different body representations interact. A body representation that relates to *where* the body is in space (Berlucchi & Aglioti, 2010; Head & Holmes, 1911; Longo, 2015a; Longo et al., 2010; Paillard, 1999) is called the postural schema, and is involved in helping guide the actions of the body (de Vignemont, 2010; Gallese & Sinigaglia, 2010). However, when considering where the body is in space no afferent signal from the body provides information about the *metric* properties of body parts (Azañón et al., 2016; Longo & Haggard, 2010) that one would need to determine the absolute location of body parts in external space (Longo, 2015a). Therefore, afferent signals regarding body orientation and joint position, such as provided under the postural schema, must be referenced to a pre-existing body representation of body size and shape (Longo & Haggard, 2010). This body representation has been termed the body model (Longo, 2015a, 2016, 2017a; Longo et al., 2010, 2015a; Longo & Haggard, 2010).

Investigators have attempted to map the body model by asking participants to localise the position of body landmarks without the use of visual information. These studies have all yielded results showing systematic distortions of the body model at the hand (Ambroziak et al., 2018; Cocchini et al., 2018; Longo, 2015b, 2017b, 2018; Longo & Haggard, 2010, 2012a, 2012b; Longo & Holmes, 2020; Longo et al., 2015b; Margolis & Longo, 2015; Mattioni & Longo, 2014; Myga et al., 2021; Peviani & Bottini, 2020; Saulton et al., 2015, 2016, 2017; Tamè et al., 2017), the forearm (Longo, 2017b), face (Fuentes et al., 2013c; Longo & Holmes, 2020; Mora et al., 2018) and leg (Stone et al., 2018).

Despite the number of studies mapping the body model, no previous study has investigated the body model of the trunk. The aim of this study was to investigate the characteristics of the implicit body model of the trunk through assessment of the perceived position sense of midline and lateral body landmarks, and then compare results to actual body positions of these landmarks.

## Methods

### Experimental Design

We conducted a cross-sectional observational study with healthy individuals within a university research laboratory. The study was approved by the Institutional Human Research Ethics Committee (Reference Number: 017188F). All participants provided signed informed consent and all procedures conformed to the declaration of Helsinki.

### Participants

A consecutive sample of 69 healthy volunteers was recruited by advertisement and word of mouth from The University of Notre Dame Australia and the local community between February 2018 and March 2019. Inclusion criteria included: aged 18–60; currently low back pain free; no history of low back pain lasting more than 24 h within the last 6 months; no low back pain requiring medical attention within the last 2 years; no other significant musculoskeletal pain (>1/10); able to stand in a stable position for up to 1 h; proficient in written and spoken English; and able to provide informed consent. Exclusion criteria included: known body perception difficulties (e.g., body dysmorphic disorder; anorexia; vestibular disorder); non-correctable visual impairment; unstable balance in standing; any current neurological, musculoskeletal, or widespread pain disorder; any significant existing medical condition; tattoo over the back which could not be suitably erased on the digital photo in Adobe Photoshop (CC 2017) (part of exclusion criteria for a concurrent study not reported here).

### Apparatus

**Figure 1.**
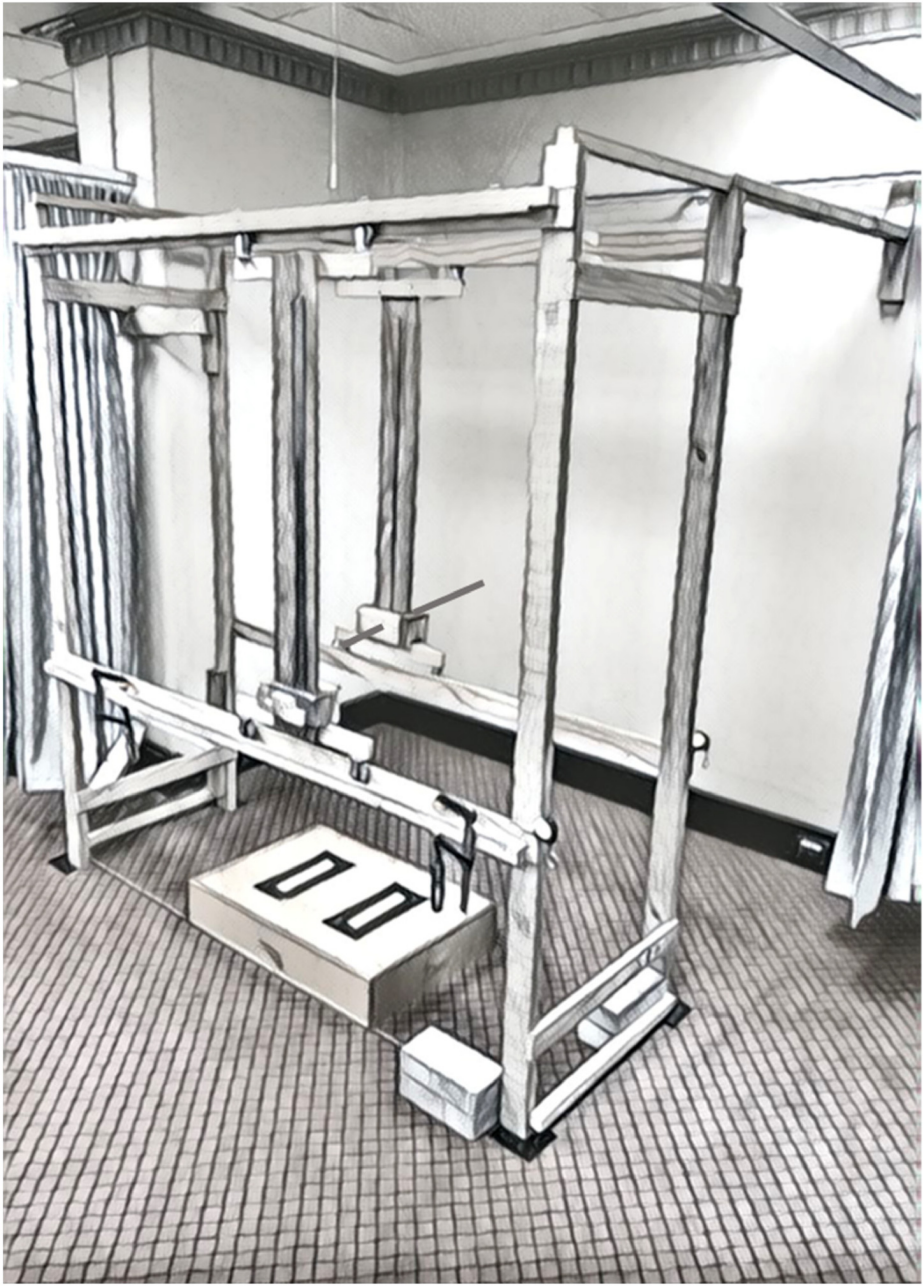
The back representation frame (BRF).

The testing device, the back representation frame (BRF) (Figure 1), consisted of a three-dimensional wooden frame 183.3 cm in length, 63.4 cm wide and 202.0 cm in height. The frame was permanently firmly anchored to the wall posteriorly and laterally. This device, and its reliability, has been previously described (Pratt et al., under review). Built into the posterior aspect of the frame was a 5 mm diameter cylindrical metal posterior pointer mounted on wheeled tracks, which an assessor could move in the horizontal (*x*) and vertical (*y*) planes (Figure 1). Using two measurement rulers permanently fixed to the frame, the *x* and *y* co-ordinates of the pointer position could be established by the assessor. When in the frame, participants were unable to see the pointer or the measurement rulers. Located in front of the participant on the anterior aspect of the frame, a matching anterior pointer was also mounted on wheeled tracks. When standing in the frame, participants could move this pointer in both horizontal and vertical directions. Similar fixed rulers enabled the *x* and *y* co-ordinates of the anterior pointer to be established.

This device allowed matching tasks to be undertaken whereby one assessor identified and measured the *x* and *y* values of the participant's body landmarks points posteriorly using the posterior pointer and could then reference this against the participant's indication of the position and level of these same body landmark points with their own pointer anteriorly, with the participant's perceived position measured by a second assessor. Similarly, for the midline localisation, the same posterior and anterior pointers were used, where the *x* values of the participant's midline could be measured posteriorly with the posterior pointer and then referenced to the participant's indication of the midline when using their own pointer anteriorly, with the participant's perceived position again measured by the second assessor. In both tasks the anterior pointer used by the participant in front of their body pointed back towards the participant's body.

### Familiarisation

Each participant was given a demonstration on the use of the BRF and provided with a period of familiarisation where they were able to practice approximately two trials of locating body landmarks with the anteriorly positioned pointer. The specific landmarks to be assessed were described to participants using a diagram of a body outline with the nine body landmarks identified. The nine body landmarks were as follows:

- Widest part of the thorax on the left
- Midline of the widest part of the thorax
- Widest part of the thorax on the right
- Narrowest part of the waist on the left
- Midline of the narrowest part of the waist
- Narrowest part of the waist on the right
- Widest part of the hips on the left
- Midline of the widest part of the hips
- Widest part of the hips on the right

### Standardised Testing Position

At the commencement of each testing session the diagonal dimensions of the frame were checked to ensure consistency across all testing sessions. A 13.5 cm high box was positioned within the frame and its position checked against markings of a string line attached to the frame. Participants stood on the box with their feet hip width apart, and in a neutral position with toes facing forward. For the final testing position, the anterior participant pointer was initially positioned 10 cm (or as close as possible to this) from the most anterior point of the participant's stomach, with the participant shuffling their feet forward or backward as required to achieve this position.

While holding the pointer at a self-selected comfortable height, the participant's shoulder was then placed in 30° abduction. This was set as the starting position for testing, with participants returning to this starting position after each repetition. To reduce external feedback, participants were instructed to make sure no part of their arm touched their body while moving the pointer and their free arm was held in slight abduction, so it wasn’t touching their body. If participants accidentally touched the side of their body, the measure was disregarded, and the trial repeated.

**Figure 2.**
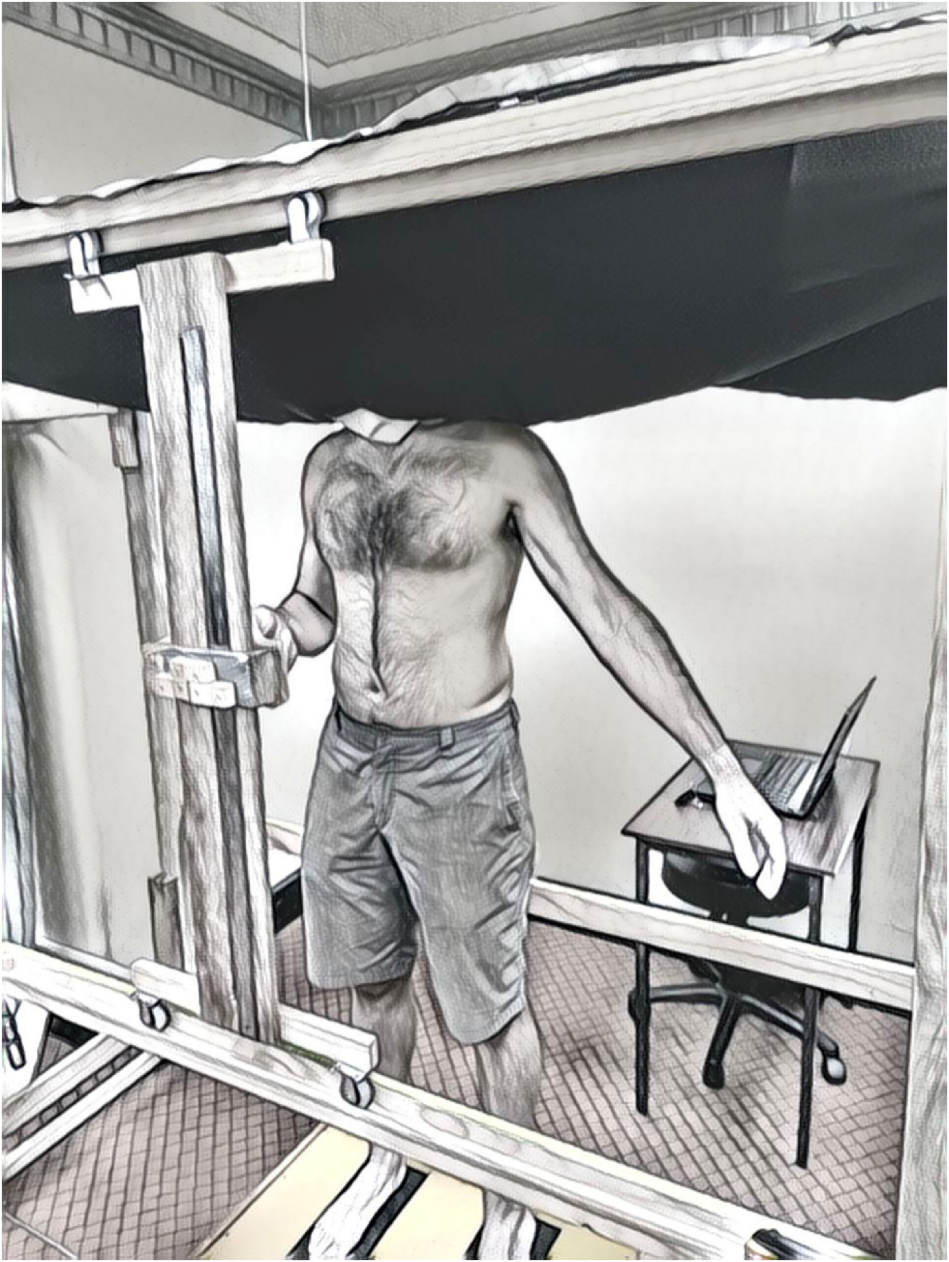
Standardised testing position.

Participants wore peripheral vision blocking goggles and a black sheet was pegged into place around their neck to ensure they could not see their body (Figure 2). In this position, participants could see the white lining of the sheet which obscured the BRF and above this, the surrounding of the room. Participants closed their eyes when moving the pointer to their respective perceived positions, however, to reduce postural sway throughout the testing period, participants were instructed to open their eyes once they were satisfied with their perceived position of each body landmark.

### Actual Midline Localisation

**Figure 3.**
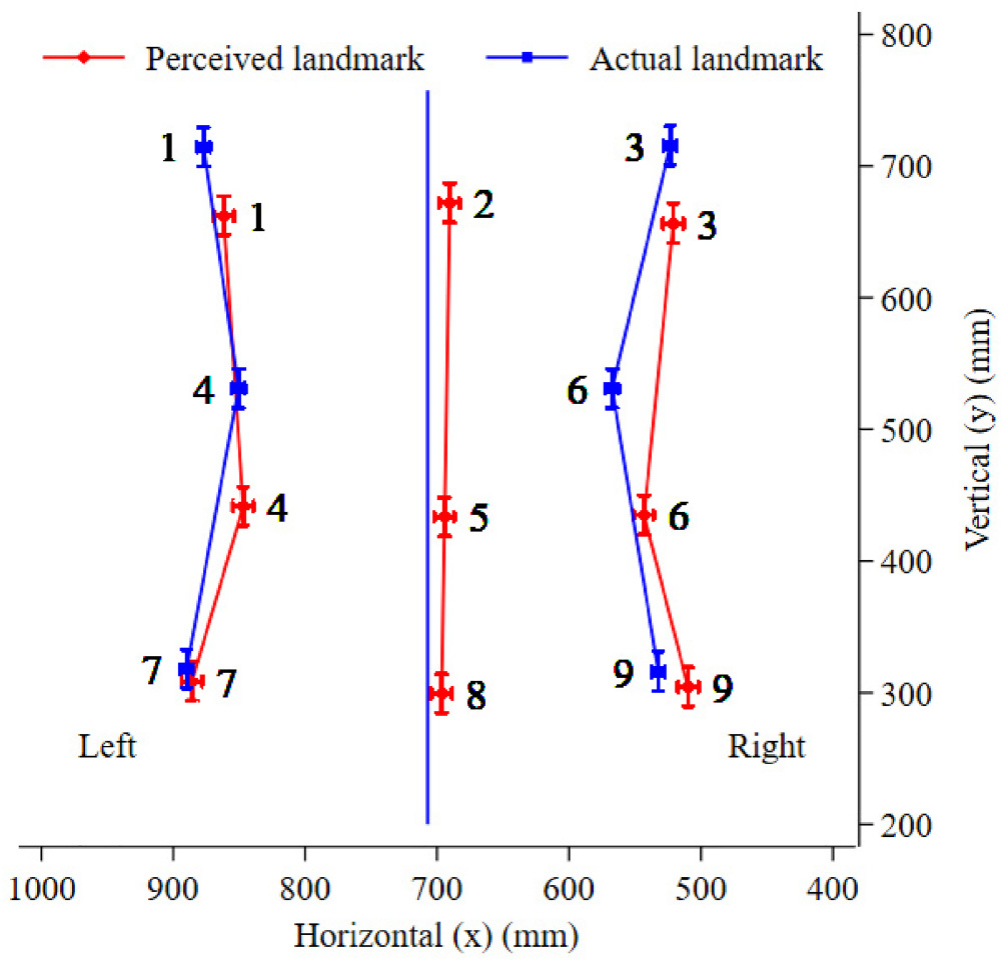
Perceived verses actual shape of the trunk (perceived verses actual *x*–*y* coordinates), outlining the perceived and actual body landmarks for the thorax (upper set of points 1–3), waist (middle set of points 4–6) and hips (lower set of points 7–9). Actual midline (not numbered) is based on the vertical projection of one midline point, taken at the lumbar spine.

To record the midline actual measure, in the standardised position, the participant performed four trials (two trials with each hand) estimating their midline at any height with the anterior pointer, having been shown a diagram of a body outline with a vertical dashed line indicating the midline (spine). This was deemed the perceived measure, but these values were not used in analysis. To determine the *x*-coordinate measure used for the actual midline line, as shown in Figure 3, the *x* and *y* co-ordinates for a spinous process at the lumbar spine was recorded for each of the four trials using the cylindrical metal pointer by the assessor positioned posterior to the participant, and the *x*-coordinate measure averaged. The actual midline line was then the vertical projection of this *x*-coordinate measure. The actual midline *y* values were only recorded at this one lumbar point, with no separate actual midline *y* values measured for the thorax, waist and hips.

### Body Landmark Localisation

For the body landmark localisation task, with the participant in the standardised position, the assessor positioned behind the participant first recorded the actual *x* and *y*-coordinates of each body landmark using the posterior pointer. Following this, the nine body landmarks were each assessed four times. The midline landmarks were assessed twice with each hand, the left hand was used for all body landmarks on the left side and the right hand was used for all body landmarks on the right side. This prevented participants receiving feedback from the arm touching the body as they crossed midline. Random allocation was first used to ascertain if tasks using the left hand or tasks using the right hand would be performed first. A computer-generated random sequence was then used to calculate the order of testing with that hand (four repetitions for the three lateral body landmarks and two repetitions for the three midline landmarks). Using the alternate hand, testing was then completed, with order determined by a separate computer-generated random sequence. The assessor positioned posteriorly had access to the random allocation sequence. They nominated each body landmark in turn and asked the participant to move the pointer to line up with where they perceived each body landmark to be. When the participant indicated they were satisfied with the position, they were asked to hold that position and the coordinates of the pointer were recorded by a second assessor positioned in front of the participant. The participant was blind to the recorded coordinates for all 36 trials.

### Variable Derivation

All analyses investigated perceived minus actual measures (in mm), where a result of zero indicated perfect agreement. For each body landmark, the actual landmark measure was subtracted from the participant's perceived landmark measure for each trial in the horizontal (*x*) and vertical (*y*) direction. A positive *x* value indicated the perceived point was to the left of the participant's actual point (i.e., the participant perceived the body landmark more to the left than it actually was) and was classified as a leftward bias. A negative *x* value indicated the perceived point was to the right of the participant's actual point (i.e., the participant perceived the body landmark more to the right than it actually was) and was classified as a rightward bias. A positive *y* value indicated the perceived point was above the participant's actual point (i.e., the participant perceived the body landmark higher than it actually was) and was classified as a superior bias. A negative *y* value indicated the perceived point was below the participant's actual point (i.e., the participant perceived the body landmark lower than it actually was) and was classified as an inferior bias.

To determine agreement in width for the lateral body landmarks at each body level, the width of the trunk was first calculated for perceived and actual, as the difference between the left and right body landmark at the thorax, waist and hip, and then the difference between perceived and actual width was calculated, again for each trial. Left and right body landmark trials were matched on order of presentation. For width, a positive value indicated the perceived width was larger than the participant's actual width (i.e., the participant perceived themselves wider than they actually are) and was classified as an overestimation. A negative value indicated the perceived width was smaller than the participant's actual width (i.e., the participant perceived themselves thinner than they actually are) and was classified as an underestimation.

For height, the distance between the thorax and hip, thorax and waist, and waist and hips were calculated for perceived and actual trials first, and then the difference between perceived and actual heights, for all trials. A positive value indicated the perceived height was greater than the participant's actual height (i.e., the participant perceived themselves longer than they actually are) and was classified as an overestimation. A negative value indicated the perceived height was less than the participant's actual height (i.e., the participant perceived themselves shorter than they actually are) and was classified as an underestimation.

### Data Analysis

Statistical analysis was performed using the Statistical Package for Social Sciences (SPSS) version 24 (IBM Corporation, New York, USA), and STATA- V17 (StataCorp. 2017. *Stata Statistical Software: Release 17*. College Station, TX: StataCorp LLC). The characteristics of participants were summarised with means and standard deviations (SDs) for continuous data and frequency/percentages for binary data.

The potential for bias in the horizontal direction was analysed using a linear mixed model which included place (left, midline or right), level (thorax, waist, hips) and the interaction between the two as fixed effects. In contrast to the models for bias in the horizontal direction, preliminary models found place and the place by level interaction were not associated with bias in the vertical direction, therefore the model used for the analyses presented here only included level as a fixed effect. Both models included place and level as crossed random effects nested within participant. Bias was inferred if the approximate Wald χ2 test for the comparison of the estimated mean bias for each body landmark to zero returned *p* < .05.

Width estimation was analysed using a linear mixed model including a fixed effect for level (thorax, waist, hips), and a random effect for level nested within individuals. Misestimation was inferred if the approximate Wald χ2 test for the comparison of the estimated mean for each width to zero returned *p* < .05.

Height was also analysed using a linear mixed model with only height components (thorax-to-hip, thorax-to-waist, and waist-to-hip) included as a fixed effect because place (left and right) and the interaction were not associated with this outcome in preliminary models. Height and place were included as a random crossed effect, nested within participant. Misestimation was inferred if the approximate Wald χ2 test for the comparison of the estimated mean for each height to zero returned *p* < .05.

All models met the assumptions of homoscedasticity and normality of residuals/random effects. We used the residual 5 SDs from the mean as a way of screening for substantially inconsistent trial values. We looked at individual points for participants, and points were set to missing if they were found to be inconsistent with all other data trials of that type. For example, for actual *x*-values of the body on the left side (mm), one participant had a thorax value of 865, a waist value of 835, however a hips value of 680, which is approximately 200 mm difference, and anatomically not likely. This number was likely transposed from 860, however it was deemed safer to set the trial to missing rather than infer a trial that was likely incorrect. All statistical hypotheses were tested against *p* < .05 and 95% confidence intervals are also presented.

The data used in this study was collected as part of a larger study therefore no *a priori* sample size calculation was conducted for these analyses. With the 69 participants included in this study, however, there is 80% power to detect a minimum bias of 14 mm at the 0.05 level, assuming a SD of 43 mm (largest observed in this study).

## Results

Ninety-eight participants were screened for eligibility, 29 were excluded for the following reasons: history of low back pain within the last 6 months (12); low back pain requiring medical attention, or lumbar spine surgery, within the last 2 years (1); known body perception difficulties (1); other musculoskeletal or widespread pain presentations (12); significant existing medical condition (2); large tattoo over the back (1). We enrolled 69 healthy participants, which included 34 males (49.3%) and 35 females (50.7%) with an average age of 29.3 years (SD = 9.5) and an average body mass index of 23.9 (SD = 3.8). Sixty-three (91.3%) participants were right-handed, and six (8.7%) were left-handed. All 69 participants completed all tasks but 0.1% (3 / 2,481) of perceived *x*, 1.2% (29 / 2,455) of actual *x*, and 1.1% (28 / 2,456) of actual *y* trials were deemed data entry errors based on residual analyses.

### Body Landmark Localisation

Figure 3 illustrates the perceived and actual coordinates for all body landmarks. This figure shows how the average body model of the trunk differs from the average actual trunk. Overall, the body model presents a squatter trunk, narrower at the thorax and wider at the waist and hips, with a rightward shift in the majority of perceived body landmarks. The observed mean *x* and *y* values containing the *x*–*y* coordinates of Figure 3, and the associated SDs, can be seen in Appendix: Table A1.

The mean bias in the horizontal direction, 95% CIs and *p*-values are presented in Table 1 for all body landmarks. Horizontal direction bias was dependent upon both the level and place co-ordinates of the landmarks assessed (level by place interaction, χ2 [4] = 160.2, *p* < .001). Bias at the thorax was similarly rightward for left lateral body and midline landmarks (see Table 1), with no evidence for a difference between the two (midline–left bias −1.7 mm, 95% CI = −10.7 to 7.3, *p* = .711). There was no evidence for bias at the thorax on the right (−2.4 mm, 95% CI = –9.9 to 5.0, *p* = .521). In contrast, the size of rightward bias increased from the left to right lateral body landmarks at the waist (right–left: −20.7 mm, 95% CI = −29.7 to −11.7, *p* < .001), and the hips (right–left: −18.5 mm, 95% CI = −27.5 to −9.4, *p* < .001). All pairwise comparisons within level and place are available in Table 2.

**Table 1.** The mean bias (mm), 95% confidence interval and associated *p*-values for the horizontal (*x*) direction mean bias values for all body landmarks.

	Left lateral body landmarks			Midline landmarks			Right lateral body landmarks		
Site	Mean bias (mm)	95% CI	*p*-value	Mean bias (mm)	95% CI	*p*-value	Mean bias (mm)	95% CI	*p*-value
Thorax	−15.1	−22.5 to −7.7	<.001	−16.8	−24.2 to −9.3	<0.001	−2.4	−9.9 to 5.0	.521
Waist	−3.5	−10.9 to 4.0	.360	−13.1	−20.6 to −5.7	0.001	−24.2	−31.6 to −16.8	<.001
Hips	−4.1	−11.5 to 3.3	.278	−10.9	−18.3 to −3.5	0.004	−22.6	−30.0 to −15.1	<.001

A negative *x*-value is classified as a rightward bias.

**Table 2.** Pairwise comparisons (defined as the value of the first comparator minus the second comparator in mm), 95% confidence interval and associated *p*-values for horizontal (*x*) direction bias values for the body landmark localisation task.

	Pairwise comparison (mm)	95% CI	*p*-value
Comparison by place (left, midline, right)			
Thorax right–thorax left	12.6	3.6 to 21.6	.006
Thorax midline–thorax left	−1.7	−10.7 to 7.3	.711
Thorax right–thorax midline	14.3	5.3 to 23.4	.002
Waist right–waist left	−20.7	−29.7 to −11.7	<.001
Waist midline–waist left	−9.7	−18.7 to −0.7	.036
Waist right–waist midline	−11.0	−20.1 to −2.0	.016
Hips right–hips left	−18.5	−27.5 to −9.4	<.001
Hips midline–hips left	−6.8	−15.8 to 2.2	.139
Hips right–hips midline	−11.7	−20.7 to −2.6	.011
Comparison by level (thorax, waist, hips)			
Waist left–thorax left	11.6	7.2 to 16.0	<.001
Hips left–thorax left	11.0	6.6 to 15.3	<.001
Hips left–waist left	−0.6	−5.0 to 3.8	.773
Waist midline–thorax midline	3.6	−0.8 to 8.0	.106
Hips midline–thorax midline	5.8	1.5 to 10.2	.009
Hips midline–waist midline	2.2	−2.2 to 6.6	.319
Waist right–thorax right	−21.8	−26.1 to −17.4	<.001
Hips right–thorax right	−20.1	−24.6 to −15.7	<.001
Hips right–waist right	1.6	−2.8 to 6.0	.475

The degree of bias in the vertical direction was only related to the level of the body landmark, that is, thorax, waist, hips (effect of level, χ2 [2] = 121.1, *p* < .001). As seen in Figure 3, there was substantial inferior bias in the vertical direction at the thorax (−56.3 mm, 95% CI = −67.6 to −45.1, *p* < .001) and waist (−91.8 mm, 95% CI = −103.1 to −80.5, *p* < .001) with no evidence for this at the hips (−10.5 mm, 95% CI = −21.7 to 0.6; *p* = .065). The waist displayed the greatest mean inferior bias of the three levels, a further 35 mm than the thorax (95% CI = 20.9 to 50.1, *p* < .001) and 81 mm more than that observed for the hips (95% CI = 66.7 to 95.8, *p* < .001).

### Width

Observed mean and SD values for perceived, actual and misestimations for width measures can be seen in Table 3.

**Table 3.** Observed mean and standard deviation (SD) values for perceived, actual and misestimations for width measures.

Site	Perceived (mm)		Actual (mm)		Misestimation (mm)	
Width	Mean	SD	Mean	SD	Mean	SD
Thorax	340.4	60.9	353.8	34.0	−12.9	55.7
Waist	300.7	59.3	283.4	31.6	20.4	50.5
Hip	374.8	49.5	357.6	30.3	16.5	47.6

Overestimation is indicated by a positive value; underestimation is indicated by a negative value.

Width misestimation was dependent upon level (χ2 [2] = 59.6, *p* < .001). On average, participants underestimated their thorax by 12.3 mm (95% CI = −24.5 to −0.2; *p* = .047). In contrast, participants overestimated their waist by 20.9 mm (95% CI = 8.7 to 33.1; *p* = .001), and their hips by 18.1 mm (95% CI = 5.8 to 30.4; *p* = .004).

There was no evidence for a difference in the magnitude of overestimation at the hips and waist (−2.8 mm; 95% CI = −12.3 to 6.6; *p* = .558). There was, however, evidence of a net difference between the underestimation of the thorax and overestimation of the waist (−33.3 mm; 95% CI = −42.6 to −23.9; *p* < .001), and the underestimation of the thorax and overestimation of the hips (−30.4 mm; 95% CI = −39.9 to −21.0; *p* < .001).

### Height

Observed mean and SD values for perceived, actual and misestimations for height component measures can be seen in Table 4.

**Table 4.** Observed mean and standard deviation (SD) values for perceived, actual and misestimations for height component measures.

	Left						Right					
Site	Perceived (mm)		Actual (mm)		Misestimation (mm)		Perceived (mm)		Actual (mm)		Misestimation (mm)	
Height component	Mean	SD	Mean	SD	Mean	SD	Mean	SD	Mean	SD	Mean	SD
Thorax-to-hip	353.9	65.4	396.8	40.9	−44.6	69.1	352.1	65.3	399.8	40.9	−48.6	73.7
Thorax-to-waist	220.9	61.5	183.7	27.2	34.0	63.3	221.4	63.9	184.2	26.7	33.1	63.1
Waist-to-hip	133.0	39.7	213.3	34.2	−79.6	54.4	130.7	41.7	214.9	35.5	−83.3	60.1

Overestimation is indicated by a positive value; underestimation is indicated by a negative value.

Height misestimation varied between thorax-to-waist, waist-to-hip and thorax-to-hip (χ2 [2] = 182.0, *p* < .001). The thorax-to-hip height was underestimated by 46.4 mm (95% CI = −60.9 to −31.9; *p* < .001), the waist-to-hip underestimated by 80.9 mm (95% CI = −95.5 to −66.3; *p* < .001) and the thorax-to-waist was overestimated by 34.1 mm (95% CI = 19.4 to 48.8; *p* < .001).

There was evidence of a net difference between the underestimation of the waist-to-hip and overestimation of the thorax-to-waist (−115.1 mm; 95% CI = −132.2 to −97.9; *p* < .001), the underestimation of the thorax-to-hip and overestimation of the thorax-to-waist (−80.5 mm; 95% CI = −97.6 to −63.4; *p* < .001) and the underestimations of the thorax-to-hip and waist-to-hip (34.6 mm; 95% CI = 17.5 to 51.6; *p* < .001).

## Discussion

The aim of this study was to investigate the characteristics of the implicit body model of the trunk via assessing the perceived position sense of midline and lateral body landmarks at the thorax, waist and hips, and then comparing results to actual body positions of these body landmarks. Significant distortion of implicit body representations of the trunk was evident. The body model presents a squatter trunk, narrower at the thorax and wider at the waist and hips. Interestingly, evidence of a rightward bias was noted in the perception of six of the nine body landmarks in the horizontal direction, which included all three levels at the midline. At the thorax, there was a similar rightward bias for left lateral body and midline landmarks, however, the size of rightward bias increased from the left to right lateral body landmarks at the waist and hips. In the vertical direction, there was evidence of substantial inferior bias in the vertical direction at the thorax and waist, with the waist displaying the greatest mean inferior bias. When considering width, at the waist and hips, there was evidence of participants perceiving themselves wider than actual width (overestimation). Conversely, at the thorax, there was evidence participants perceived themselves smaller than actual width (underestimation). For height, on average, participants perceived the trunk shorter (underestimation) at the thorax-to-hip and waist-to-hip heights, and longer (overestimation) at the thorax-to-waist height, with the largest difference being at the waist-to-hips height, resulting in overall, participants perceiving a squatter representation of their trunk. Interestingly, the degree of inaccuracy from the mean difference of the perceived minus actual measures was a great deal larger in the height direction, compared to the width direction. This may be reflective of both having to use knowledge of our trunk width much more frequently than considering trunk height when negotiating with our surroundings, and at the trunk, clearer lateral body borders for width reference.

Considering the midline landmarks, our study reported a rightward bias. Findings from midline perception studies, where healthy participants estimate perceived midline by stopping an illuminated dot or by pointing straight ahead, have been somewhat ambiguous. Participants have been shown to demonstrate a leftward shift in their visual subjective body midline (Farnè et al., 1998; Reinersmann et al., 2012; Sumitani et al., 2007), no or virtually no deviation (Sumitani et al., 2007; Verfaille et al., 2021), and a rightward shift (Kolb et al., 2012), although it is unclear if the shift reported by Kolb et al. (2012) and Farnè et al. (1998) is of significance. The rightward shift in the current study contrasts with a previous review of literature reporting a leftward shift in mid-sagittal pointing tasks (Jewell & McCourt, 2000). A leftward shift in midline perception has been argued due to the right-hemisphere dominance in visuospatial (Reinersmann et al., 2012) and egocentric processing (Zhou et al., 2017), and the potential greater representation of the ipsilateral visual field in the right hemisphere compared to the left (Zhou et al., 2017). Our study found a slight rightward bias in midline landmark perception (and indeed for the majority of body landmarks in the horizontal direction), in line with Kolb et al. (2012) where participants eyes were also closed, and so this rightward shift may also be due to participants performing the task with no visual input. This rightward bias is opposed to the majority of the above studies, where participants could use their vision, and thus further engage the right hemisphere, potentially causing a leftward shift in midline as suggested above, with the relationship between the right hemisphere and vision, when visual input is used (Reinersmann et al., 2012; Zhou et al., 2017). A further fundamental methodological difference in the current study that may explain differences with results from previous studies, centres on participants relying on an internal reference system of their body to identify body landmarks and estimate midline on their *own* body, as opposed to predicting midline via an illuminated dot or pointing straight ahead, which may be argued to link midline predictions to an external point to the body. The influence of hand dominance may also play a role in this study's observed midline results. When asking participants to point straight ahead of their perceived body midline without visualising their hand, healthy controls made errors toward their dominant side when pointing with their dominant arm, and toward their non-dominant side when pointing with their non-dominant arm (Vittersø et al., 2021). With the majority of participants in the current study right-hand dominant, this may explain the rightward midline bias observed in our study (and also the rightward bias in the majority of body landmarks). However, with the same point straight-ahead task, participants have also been shown to demonstrate a non-significant leftward deviation (Chokron & Imbert, 1995), and a subsequent study also reported a leftward deviation (Farnè et al., 1998), although the significance of this deviation was not reported, and the effect of hand dominance was not reported for either study. With only six left-handers in this study, analysis was not conducted for handedness, as any effect could not be established as reliable with such low numbers.

Midline perception has also been investigated in patients with pain or neurological conditions, which would be the next step for this current research. As with healthy participants, results for patients with chronic pain are variable. Participants with complex regional pain syndrome (CRPS) did not demonstrate any midline deviation when performing a point straight ahead task for midline either blindfolded (Verfaille et al., 2021) or in dark conditions (Christophe et al., 2016), however with eyes closed, another study found participants deviated to the same side as the arm used (Vittersø et al., 2021). With the visual task of stopping an illuminated dot at the perceived midline position, in illuminated conditions, results also demonstrated no midline deviation for patients with CRPS (Sumitani et al., 2007; Uematsu et al., 2009; Verfaille et al., 2021) and patients with post-herpetic neuralgia (Uematsu et al., 2009). However, in dark conditions, results for participants with CRPS have varied from no perceived midline deviation (Christophe et al., 2016; Verfaille et al., 2021), to a perceived midline shift to the affected side (Sumitani et al., 2007; Uematsu et al., 2009), or toward the left, regardless of affected side (Reinersmann et al., 2012). Investigations with patients in chronic upper limb pain have also shown a shift in perceived midline toward the affected side (Reinersmann et al., 2012). In studies comparing healthy participants and patients with CRPS, no significant difference was found in midline perception for tasks where participants pointed ahead with vision obstructed (Kolb et al., 2012; Verfaille et al., 2021; Vittersø et al., 2021) or directed an illuminated dot/rod to their perceived midline in light (Verfaille et al., 2021) and dark conditions (Verfaille et al., 2021; Wittayer et al., 2018). In contrast, participants with CRPS have also demonstrated a significantly larger leftward shift in their perceived midline compared to patients with chronic upper limb pain and healthy controls (Reinersmann et al., 2012).

The finding in this study of greater error in the vertical direction is largely consistent with previous studies of tactile function that demonstrated distortions in distance perception. When assessing vibrotactile stimulation, distances were perceived as longer in the vertical direction compared with the horizontal direction at the thoracolumbar spine (Plaisier et al., 2020), and at the lower thoracic spine, higher inaccuracy was recorded for vertical presentation of stimuli compared with horizontal (Hoffmann et al., 2018). A further study assessing pressure stimuli to the lower thoracic spine found distances presented vertically were overestimated compared to horizontally (Nicula & Longo, 2021). Assessing vibrotactile stimulation at the thoracic spine, results have shown reduced accuracy in the vertical axis for a direction discrimination task (Jouybari et al., 2021). Of note however, is the contrasting findings from pressure stimuli applied at the upper back (centre of scapula), with stimuli applied across the body width overestimated compared to along body height (Nicula & Longo, 2021). Furthermore, at the abdomen, although patients with anorexia nervosa have been shown to overestimate the distance between two tactile stimuli compared to a control group (Keizer et al., 2011, 2012) and judge horizontal tactile stimuli as wider relative to the same stimuli applied vertically compared to a control group (Spitoni et al., 2015), a lack of evidence of difference was reported when perceived distance was assessed along and across the belly in a separate study of women with no tactile abnormalities at the belly (Longo et al., 2019). Despite these varied results, distortions in perceived distance with tactile function have been suggested to be due to varying receptor field densities and shapes (Longo & Haggard, 2011; Plaisier et al., 2020) and varying cortical representations for different skin regions (Taylor-Clarke et al., 2004).

Our findings on width and height are consistent with previous research from studies where the body part was obscured, and participants asked to indicate the spatial location of defined points. Previous studies show, despite assumptions that healthy adults have accurate representations of their body, large and highly stereotyped distortions of mental body representations are apparent (Longo, 2017a, 2022; Longo & Holmes, 2020). The overestimation of width at the waist and hips aligns with previous findings of overestimation in the medio-lateral direction at the hand (Longo, 2017b, 2018; Longo & Haggard, 2010, 2012a, 2012b; Longo & Holmes, 2020; Longo et al., 2015b; Mattioni & Longo, 2014; Peviani & Bottini, 2020; Saulton et al., 2015, 2016, 2017; Tamè et al., 2017), forearm (Longo, 2017b), face (Longo & Holmes, 2020; Mora et al., 2018), leg (Stone et al., 2018), and waist/hip (Hach et al., 2011). Earlier studies that were based around the image marking procedure first adopted by Askevold (1975), where participants stand and are touched at certain points on their body (e.g., chest, waist, hips, thighs) and then mark on a board the perceived positions of these points (Lautenbacher et al., 1992), also reported overestimation of body dimensions in healthy participants (Meermann, 1983), including the waist and hips (Thomas & Freeman, 1991). Furthermore, in a study where vision was not obscured and participants drew where the perceived location of a named body part on an image of their own body, results found when comparing arm length/shoulder width and leg length/hip width participants also represented the body as wider (overestimation of width), relative to limb length, than it actually was (Fuentes et al., 2013b). A subsequent study using the same methodology also reported participants overestimated the width of the torso, although participants also overestimated torso length, which differs from the current study (Fuentes et al., 2013a). For height, if considering the trunk overall with thorax-to-hips height, participants tended to underestimate their trunk in the vertical axis, consistent with previous findings of underestimation in length or proximo-distal direction, for the hand (Cocchini et al., 2018; Longo, 2017b, 2018; Longo & Haggard, 2010, 2012a, 2012b; Longo & Holmes, 2020; Longo et al., 2015b; Mattioni & Longo, 2014; Myga et al., 2021; Peviani & Bottini, 2020; Saulton et al., 2015, 2016, 2017; Tamè et al., 2017), face (Fuentes et al., 2013c) and leg (Stone et al., 2018).

The source of these distortions is unclear, with some research suggesting it may be due to anisotropies and distortions of somatosensory cortical maps (Longo & Haggard, 2010; Longo et al., 2015a, 2015b), while other research suggests distortions may be due to memory and perceptual processes (Medina & Duckett, 2017; Saulton et al., 2015). Medina and Duckett (2017) argue distortions are not due to a distorted body representation, but rather are due to uncertainty when estimating consecutive stimuli close in space, but difficult to localise. However, our study discovered distortions despite body landmarks not being close in proximity. Interestingly, previous research suggested that somatosensory components (i.e., tactile acuity) and visual components (i.e., demarcation of visual landmarks) contribute to distortions, with underestimation in body parts that were visually accessible (hand, foot and lips), yet accurate estimation for the dorsal neck, indicating a metric bias may occur when visual information is weighed more heavily in developing the mental representation (Peviani et al., 2019). Our results show conflicting results with these findings, with accuracy varying across the trunk: participants’ width misestimation was a great deal lower (more accurate) than height misestimation. Peviani and Bottini (2020) argue hand distortions may be due to a complex interaction between cortical representation and other influences relating to structural information of the body such as proprioceptive and spatial memory biases. Longo et al. (2015b) suggested distorted body representations in the size and shape of the hand were due to both perceptual distortions, where there is spatial warping of the representation of body tissue that may reflect distortions of somatotopic maps, and conceptual distortions, due to incorrect beliefs of the location of different body landmarks (Longo, 2017a; Longo et al., 2015b), which also may explain the inferior bias observed in the vertical body landmarks. Longo (2015b) and Saulton et al. (2017) also propose distorted conceptual knowledge of hand structure may (at least partly) explain distortions observed at the hand. Interestingly, when assessing the haptic perception of parallelity where blindfolded participants rotated a test bar in front of their body to be parallel to a reference bar, the size of deviations measured were dependent on the weighting of egocentric and allocentric reference frame involvement, where the egocentric reference is a reference frame linked to the body or specific body part (i.e., hand and arm orientation), and allocentric is independent of the actual position of the perceiver and related to external space and the position of the body in relation to the surrounding environment (Volcic et al., 2007). Research has also shown that participants display larger errors when pointing forwards to project their body (allocentric representation) compared to pointing backwards to their occluded waist and hips (egocentric reference frame) (Hach et al., 2011). Indeed, the effect and weighting of reliance on egocentric and allocentric reference frames could influence our results and be a topic of further study.

While this is the first known investigation of the body model of the trunk, there have been a number of studies investigating body image, an explicit body representation, of the lumbar spine (Moseley, 2008; Nishigami et al., 2015; Wand et al., 2014, 2016). These investigations have been focused on people with low back pain and a consistent finding from the application of self-reported measures of body size and shape is that people with low back pain perceived their back to be enlarged (Wand et al., 2014, 2016). Just like applying an anaesthetic to a body part results in reduced sensory input and a feeling of enlargement to the area (Gandevia & Phegan, 1999), one explanation behind the feeling of enlargement for people with chronic low back pain may be due to the degradation of proprioceptive input (Wand et al., 2023) and tactile acuity (Adamczyk et al., 2018) which occurs with this condition. These findings may also suggest a potential association between the implicit body representation of the body model, and the explicit body representation of the body image.

This study has some limitations to consider. The estimation of midline and lateral body landmarks was performed as part of a longer two-hour testing session, and with this task performed approximately mid-way through the session, participant concentration may have been a factor in performance. Future studies could look to utilise this method in a stand-alone assessment session. Each of the body landmarks were estimated four times, however, increasing the number of trials may help for a further robust data set to analyse for findings. In terms of hand dominance, there weren’t enough left-handed participants to investigate the effect of handedness in comparison with right-handed participants. Previous research has shown an effect of hand dominance when participants were asked to point to their shoulder, waist or hips, occluded from view (Hach & Schütz-Bosbach, 2010). Right-handers displayed an asymmetric estimation of their body, pointing at a greater distance to the midsagittal plane in right hemispace when compared to pointing in left hemispace; this asymmetry was not present for left-handers (Hach & Schütz-Bosbach, 2010). In a subsequent study, right-handers were shown to display significantly larger errors when pointing to the waist and hips in the right hemispace compared to the left hemispace, a finding not seen with left-handers (Hach et al., 2011). The effect of hand dominance therefore warrants further investigation.

### Conclusion

This study demonstrates evidence of a significant distortion of implicit body representations of the trunk, with participants demonstrating evidence of a significant difference when comparing perceived to actual location in the horizontal (*x*) and vertical (*y*) directions for the majority of trunk landmarks. In the horizontal direction, evidence of a rightward bias was noted in the perception of six of the nine body landmarks, including all midline levels. In the vertical direction, at the thorax and waist, a substantial inferior bias in the *y* direction was evident, being greatest at the waist. Considering width and height, the body model presents as a squatter trunk, wider at the waist and hips. These results show some similarities to previous research which has demonstrated overestimation from participants in the medio-lateral direction, and underestimation in the proximo-distal direction. Furthermore, the larger inaccuracies seen with height compared to width values may be reflective of the higher need to negotiate width compared to height for everyday function of the body in negotiating its surroundings, and for the trunk, more defined lateral body borders for width reference.

## Appendix

**Table A1.** Body landmark task: Observed mean and standard deviation (SD) values for *x*- and *y*-coordinates.

	Left				Middle				Right			
	Perceived *x* (SD)	Actual *x* (SD)	Perceived *y* (SD)	Actual *y* (SD)	Perceived *x* (SD)	Actual *x* (SD)^a^	Perceived *y* (SD)	Actual *y* (SD)^b^	Perceived *x* (SD)	Actual *x* (SD)	Perceived *y* (SD)	Actual *y* (SD)
Thorax	861.8 (38.1)	876.9 (21.1)	662.3 (71.2)	714.0 (68.0)	690.5 (28.4)	707.1 (18.6)	672.2 (73.4)	—	521.0 (39.2)	523.4 (27.8)	656.4 (75.8)	715.0 (68.2)
Waist	847.0 (37.2)	850.4 (21.0)	441.5 (55.1)	531.4 (57.5)	694.4 (24.7)	707.1 (18.6)	433.5 (50.7)	—	543.2 (33.5)	567.3 (23.8)	435.0 (55.8)	531.2 (57.8)
Hips	885.8 (36.4)	889.9 (23.5)	308.5 (59.3)	317.8 (49.2)	696.6 (26.4)	707.1 (18.6)	299.4 (57.2)	—	509.8 (33.2)	531.7 (21.6)	304.3 (62.1)	317.0 (48.9)

a Actual midline *x* values are based on the vertical projection of one midline point, taken at the lumbar spine.

b As the actual midline *y* values were only recorded at one lumbar point, no separate actual midline *y* values were measured for the thorax, waist and hips.
